# Effects of hydroquinone-containing creams on capillary glycemia before and after serial hand washings in Africans

**DOI:** 10.1371/journal.pone.0202271

**Published:** 2018-08-28

**Authors:** Simeon-Pierre Choukem, Derrick Tembi Efie, Sefirin Djiogue, François F. Kaze, Yannick Mboue-Djieka, Thadée Boudjeko, Etienne Dongo, Jean-François Gautier, Andre-Pascal Kengne

**Affiliations:** 1 Department of Internal medicine and Paediatrics, Faculty of Health Sciences, University of Buea, Buea, Cameroon; 2 Health and Human Development (2HD) Research Network, Douala, Cameroon; 3 Department of Internal Medicine, Douala General Hospital, Douala, Cameroon; 4 Department of Animal Biology and Physiology, Faculty of Science, University of Yaounde I, Yaounde, Cameroon; 5 Department of Internal Medicine and Subspecialties, Faculty of Medicine and Biomedical Sciences, University of Yaounde I, Yaounde, Cameroon; 6 Department of Biochemistry, Faculty of Science, University of Yaounde I, Yaounde, Cameroon; 7 Department of Organic Chemistry, Faculty of Sciences, Universityof Yaounde I, Yaounde, Cameroon; 8 Department of Diabetes, Endocrinology and Nutrition, Assistance Publique - Hôpitaux de Paris, Lariboisière Hospital, University Paris-Diderot Paris-7, Paris, France; 9 South African Medical Research Council and University of Cape Town, Cape Town, South Africa; Medical University of Vienna, AUSTRIA

## Abstract

**Background:**

Hydroquinone-containing creams cause false increases in capillary glycemia. However, the magnitude of this false increase, and the means to reverse it have not been investigated.

**Objective:**

To evaluate the technical and clinical impact of hydroquinone-containing creams on capillary glycemia and investigate the efficacy of hand washing and other common practices, in reversing cream effects.

**Methods:**

We included 91 participants in a quasi-experimental study in Buea, Cameroon. After determining the hydroquinone content of a cream, Caro Light, we used two glucometers with different enzymatic systems (Accu-Chek Active and OneTouch Ultra 2) to measure fasting glycemia after: initial hand washing (reference), application of 1 ml of hydroquinone-containing cream, finger swabbing with wet gauze, sanitizer application and a series of three hand washings following cream application. Reference glycemia was compared to those obtained after various interventions. Statistical significance was assessed by paired sample t-test, clinical significance by total error allowable (TEa), and clinical impact by Parke’s error grid analysis.

**Results:**

The mean differences in capillary glycemia (Intervention—reference) measured by Accu-Chek Active in mg/dl were 28, 27, 38, 16, 4, and -2 after cream application, finger swabbing, sanitizer application, one, two, and three hand washings respectively. Corresponding values for OneTouch Ultra2 were 41, 44, 64, 22, 5 and -5. These differences, except after two and three hand washings were both statistically (p < 0.0001) and clinically significant (TEa). After cream application, Accu-Check had 9.9% of values in Parke’s Zones C-E, while OneTouch had 18.7%.

**Conclusion:**

Hydroquinone-containing creams cause significant false increase in capillary glycemia irrespective of the enzymatic system of the glucometer used, and can lead to potentially wrong clinical decisions. A minimum of two hand washings is required prior to capillary glucose measurement.

## Introduction

Point-of-care(POC) glucometers have widely been adopted as tools to provide timely feedback for diabetes management and monitoring, and for diabetes risk screening and diagnosis in resources-limited and remote settings [1,2]. Issues with the accuracy of the measurements can severely affect decision-making based upon glucometer-acquired blood glucose levels [3]. Accuracy is a measure of how closely the average of a set of values reflects the reference value. Four main factors alter the accuracy of glucometers: strip factors, physical or environmental factors, pharmacological factors and patient factors [3]. The latter includes incorrect hand washing or interfering substances such as hand lotions, alcohol, or dirt.

Errors in blood glucose measurement due to extrinsic interfering substances continue to significantly plague the accuracy of results. This has major clinical impact especially with the indiscriminate use of over-the-counter creams for skin bleaching [4]. Hydroquinone (Benzene- 1,4-diol), a bleaching agent found in skin lightening creams has been reported to falsely elevate capillary glucose measurement [5–7]. In a study involving 11 healthy volunteers in France, we showed that hydroquinone-containing creams caused a seeming mean increase in capillary glycaemia of 170 mg/dl [5]. This study however did not evaluate the clinical significance of the increase in capillary glycaemia due to the cream.

Opinions diverge on whether to implement hand washing or hand disinfection prior to capillary glucose measurement [8]. Although the importance of proper hand washing prior to capillary glucose measurement (CGM) cannot be overemphasized, it is not a routine clinical practice to observe hand washing before CGM [1]. The 2015 International Diabetes Federation guidelines recommends at least one hand washing prior to capillary glucose measurement. Hortensius and colleagues showed that only 31% of patients with diabetes who self-monitor their blood glucose wash their hands before measurement [8]. They also reported a mean capillary glucose level after hand exposure to a fruit (apple or banana) of 270 mg/dl, which dropped to 160.2 mg/dl after hand washing once with soap and water [9]. Existing studies however do not provide any data on the actual minimum number of times hands should be washed prior to CGM. Little is known about the clinical impact of hydroquinone-containing creams on CGM as well as means to reliably reverse these effects in routine settings.

We assessed the effect of hydroquinone-containing body creams on the accuracy of two glucometers with different enzyme principles, and investigated if finger swabbing, application of hand sanitizer or serial hand washings could reverse these effects in a population of Cameroonians with or without diabetes.

## Methods

### Study design, setting and population

We included 91 consenting consecutive participants with and without diabetes in a quasi-experimental study carried out in Buea, Cameroon. Participants were in fasting state and were asked about any recent intake of common substances that could interfere with capillary blood glucose measurement. The selection of glucometers was restricted to those labelled for self-monitoring of blood glucose, carrying a European Conformity mark, certified ISO 15197:2013, and licensed for marketing in Cameroon. Therefore, the following two glucometers using different biochemical principles were selected: OneTouch Ultra2 (glucose oxidase enzymatic principle) and Accu-Check Active (glucose dehydrogenase enzymatic principle). In total, two glucometers were used throughout the study. One Accu-Check Active glucometer and one OneTouch Ultra 2. We used strips with Lot Number 24638031 for Accu-Check Active, and number 17B06 for OneTouch Ultra 2. The features of the two glucometers, and the recommended environmental conditions of use are presented in Table 1.

**Table 1:** Glucometers used and their characteristics.

	Accu-chek Active	OneTouch Ultra 2
Manufacturer	Roche Diagnostics	Life Scan
Enzyme System used	Glucose Dehydrogenase	Glucose oxidase
Test strip	Accu-Chek active test strips	One touch ultra-strips
Coding	Automatic via code chip	Manual input
Test time	5 seconds	5 seconds
Sample volume	1–2 μL	0.5 μL
Operation temperature	8°–42°C	6°–44°C
Operation Humidity	10–85% Relative Humidity	10–85% Relative Humidity
Measurement calibration	Plasma equivalent	Plasma equivalent
Test method	Photometry	Photometry

Both glucometers used in the study met the ISO 15197:2013 guidelines which states that ninety-five (95%) of the individual glucose results should fall [10]:

- Within ±15% of the results of the manufacturer’s measurement procedure at glucose concentrations < 75 mg/dl (Within ± 0.83 mmol/L of laboratory results at concentrations of under 4.2 mmol/L)
- Within ±20% at glucose concentrations ≥ 75 mg/dl (4.2 mmol/L)

Ethical approval was obtained from the Institutional Ethics Committee for Research on Human Health of the University of Douala and written informed consent was obtained from all participants prior to inclusion.

### Confirmation of the hydroquinone content in the cream

Caro Light cream label indicated 2% of hydroquinone. Determination of actual hydroquinone concentration in the cream followed the protocol reported by Garcia et al., 2007 [11]. One gram of cream was dissolved in 20 mL of sulfuric acid 0.05 M (Carlo Erba Reagents, Val de Reuil, France) in a *bain-marie* (Precisterm Selecta) at 40°C. The solution obtained after cooling was filtered with Whatman paper. Absorbance was read by a spectrophotometer (Shimadzu 1605, Tokyo, Japan) at 302 nm. The precision of this spectrophotometer in this wave length is **±** 2 nm and the reproductibility is **±** 0.5 nm. The concentration was then determined from a calibration curve obtained from pure hydroquinone (Xi’an Lyphar Biotech, China) at various concentrations (6.25, 12.5, 25, 50 and 100 μg/mL). The test was repeated six times. The concentration of hydroquinone in the cream was 1.55 ± 0.018 milligrams per gram of cream (0.155%), which is thirteen-fold lower than the labelled concentration.

### Study procedures and data collection

Participants were evaluated in the morning after an overnight fast of at least 8 hours prior to the study procedures. For each participant, serial capillary blood glucose concentrations were measured with the two glucometers under investigation, following a series of interventions. Calibration of the glucometers using control solutions and test strips was carried out at the start of each day. Study procedures included three sets of consecutive interventions, with each subsequent intervention implemented within 5 minutes of completing the previous intervention. Interventions and capillary glucose measurements (hand side chosen by the participant) were performed on different fingers so as to avoid any interaction between them (S1 Fig).

The procedure started each day with the calibration of the glucometers. We then proceeded with the interventions on participants, as follow:

- The investigator washes and dries his own hands, then puts on gloves after having assembled the materials needed for the procedure.
- The procedure to be performed is explained to the participant.
- The participant washes his/her hands thoroughly with soap and water and dries them with a clean towel, all under the observation of the investigator.
- The strip box is opened and the test strip inserted into the meter as per manufacturer’s instructions.
- The participant’s finger is pricked with a lancet as per manufacturer’s instructions, and the blood drop is applied to the test strip, at baseline and after each intervention. The test results display automatically on the screen of the device.
- A piece of gauze is applied to the participant’s finger at the pricked site and gentle direct pressure is applied.
- The participant’s capillary blood glucose result is recorded on the case report form.
- Used lancets are placed into disposal containers immediately.
- Used test strips are disposed into clinical waste bin before gloves are removed
- The external surfaces of the meter are cleaned with an alcohol based wipe and the meter is ready to be used again.

All glucose measurements were made at room temperature between 14 and 27°C, with a relative humidity of 75% to 85%, which all fall within the prescribed operating temperature and humidity for the two glucometers used in the study (Table 1).

#### Reference glycemia

Participants were asked to wash their hands once with soap and running water for about two minutes and dried them. A finger was then pricked with a sterile lancet and capillary glucose measured using the two hand-held glucometers under investigation. The values obtained served as reference capillary glucose values for each participant.

#### Intervention 1

Using a calibrated syringe, 1ml of Caro Light (hydroquinone-containing cream) was applied to the participant’s palm and the hands rubbed gently and uniformly against each other for about 10 seconds. The cream was allowed to set for 60 seconds, after which capillary glucose concentration was measured from a cream exposed finger using the 2 glucometers.

#### Interventions 2A and 2B

A cream-soiled finger was swabbed once with sterile water-soaked gauze (2A). At the same time, on another cream-soiled finger, an instant hand sanitizer was applied to the finger and briskly rubbed, covering the finger with the product until it dried (2B). After these procedures, capillary glycemia was measured from both fingers using both glucometers.

#### Intervention 3

The final intervention consisted of a series of three hand washings with neutral soap and running water. Each hand washing lasted about two minutes and hands were dried thereafter. Capillary glycemia was measured after each hand washing from a finger that had previously been exposed to the skin lightening cream but had neither been swabbed with wet gauze nor exposed to hand sanitizer.

Seven (7) glucose measurements were performed per glucometer per participant, giving a total of 14 measurements per participant. The 7 measurements were: 1) after hand washing but before cream application (reference glycemia), 2) after hydroquinone cream application (intervention 1), 3) after finger cleaning with wet gauze (intervention 2A), 4)after sanitizer application (intervention 2B), and three more measurements respectively after the first, second and third hand washing following cream application (intervention 3).

### Statistical analysis

Data analysis used R statistical software version 3.2.2 (The R Foundation for Statistical Computing, Vienna, Austria). Variables are summarized as means (standard deviation), median (min-max), and count (percentages). The Shapiro-Wilk W test was used to determine whether the glucose measurements were normally distributed, based on probability thresholds of p≥0.1. The student *t-test* was used to compare characteristics across gender and diabetes status. Glucose measurement after the initial hand washing and before cream application served as the reference for all comparisons between two measurements. The continuous association between glucose measurements was assessed using Pearson’s and Spearman correlations. Paired-sample *t-test* was used to compare differences in means of glucose measurements overall and within subgroups. The threshold for statistical significance was set at 0.05.

To assess the clinical importance of statistically significant differences between post-intervention glucose measurements and the reference measurement, Parke’s error grid analysis and total error allowable (TEa) [12,13] were used. The mean of each glucometer measurement was then compared with the reference and should be within the clinical range of the reference mean ± TEa. Here, we considered the TEa for glucose to be 6.96 [14]

### Ethical approval

Ethical approval was obtained from the Institutional Ethics Committee for Research in Human Health of the University of Douala, and administrative clearance from the Director of the Buea Regional Hospital. Written informed consent was signed by all participants.

## Results

### General characteristics of participants

Of the 91 participants included, 37 (41%) had diabetes. The age of the participants ranged from 22 to 75 years. Other general characteristics are shown in Table 2.

**Table 2:** General characteristics of the participants.

Variables	Overall	No diabetes	Diabetes	p-value	Men	Women	p
N (%)	91 (100)	54 (59)	37 (41)		44 (48)	47 (52)	
Men, n (%)	44 (48)	24 (44)	20 (54)	0.368			0.951
Mean age, years (SD)	44.1 (16.3)	34.7 (11.3)	57.9 (12.0)	<0.0001	42.5 (16.3)	45.6 (16.3	0.371
Mean BMI, kg/m^2^ (SD)	27.8 (6.8)	28.6 (7.7)	26.6 (5.3)	0.151	25.3 (5.8)	30.1 (7.0)	0.0006

BMI: Body mass index; SD: Standard deviation

### Profile of capillary glycemia

**Fig 1:**
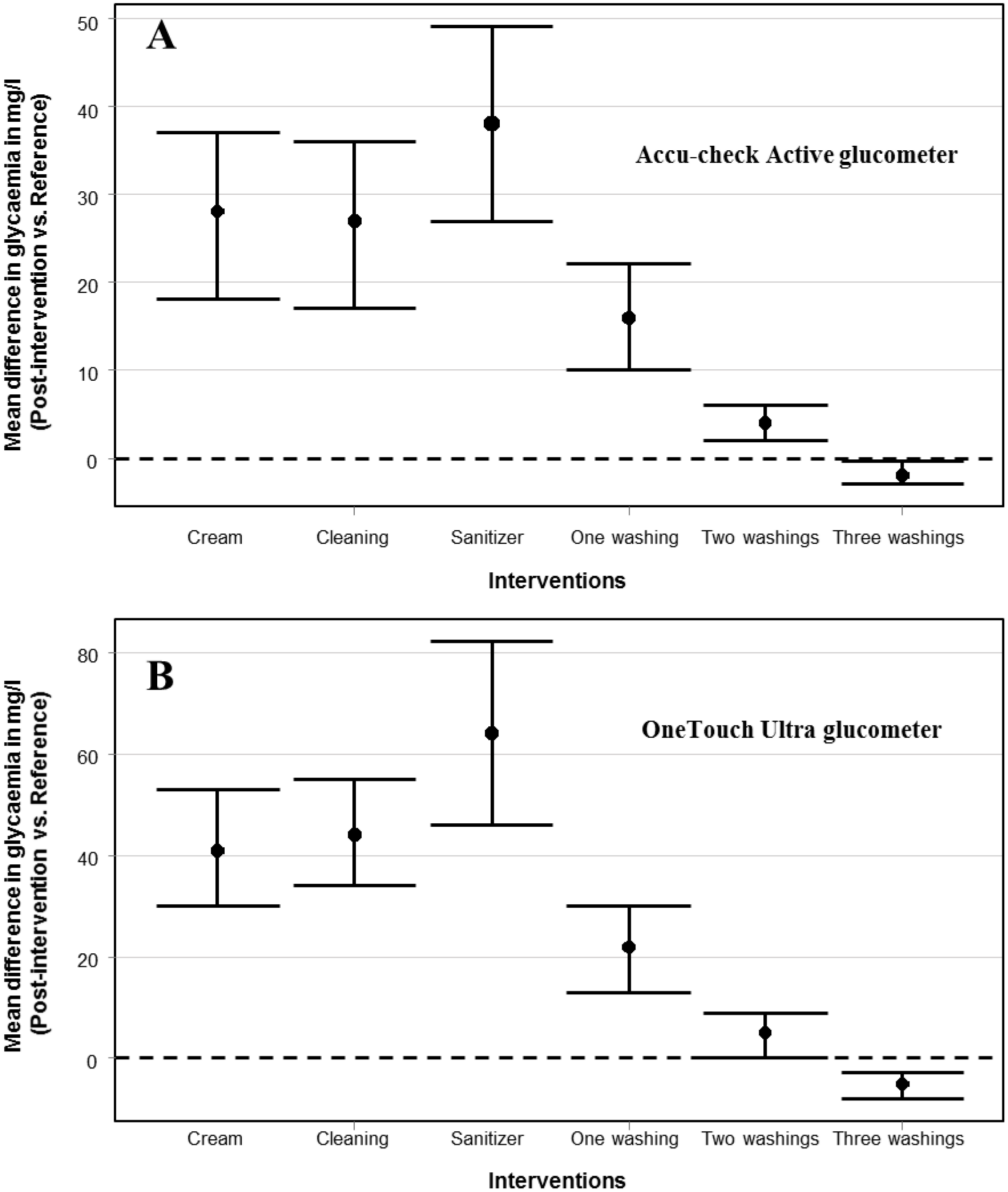
The mean differences in capillary glucose between reference values and those obtained after various interventions in the general population. 1A: Accu-check Active; 1B: OneTouch ultra2.

The baseline reference capillary glucose (in mg/dl) ranged from 50 to 452 (mean 134; median 95) using the Accu-Check Active glucometer, and from 51 to 409 (mean 138; median 100) using the OneTouch ultra 2 glucometer. The profile of capillary glucose for the whole populationis is shown in Fig 1(A) and 1(B). S1 Table (Accu-Check Active) and S2 Table (OneTouch Ultra 2) further depict details per subgroups (men and women, diabetes and no diabetes). Overall and in subgroups, capillary glucose rose after cream application, remained stable after finger cleansing, futher increased after application of hand sanitizer, then decreased progressively after each hand washing to return to or slightly below the baseline value after the third hand washing.

### Effect of hydroquinone-containing cream on capillary glycemia

**Fig 2:**
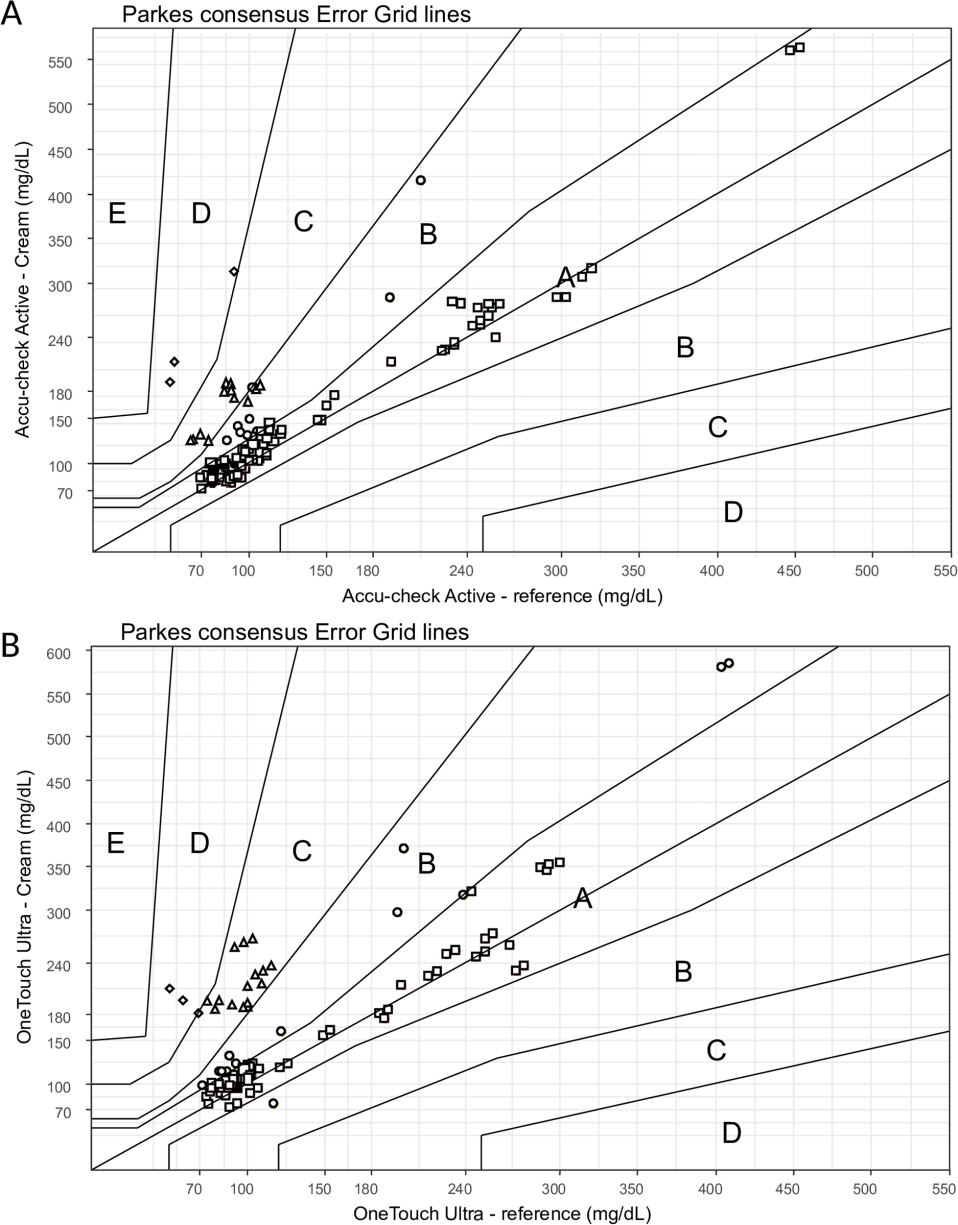
Parke’s error grid analysis comparing the reference capillary glucose measurement against the value obtained after hydroquinone-containing cream application. 2A: Accu-check Active; 2B: OneTouch ultra2. Open squares are in zone A, open circles in zone B, open triangles in zone C and open diamonds in zone D.

Capillary glucose values obtained after hydroquinone-containing cream application were consistently higher than reference values across the two glucometers overall, in men and women and in participants with and without diabetes (all p < 0.0001). The mean difference (post-cream values minus reference values) was 28 (95%CI: 18 to 37) mg/dl for Accu-check Active (S1 Table), and 41 (95% CI: 30 to 53) mg/dl for OneTouch ultra2 (S2 Table). These differences were clinically significant in the overall population, and across gender and diabetes status based on TEa (Table 3). In Parke’s consensus grids (Fig 2), 9.9% of Accu-check Active post-cream values and 18.7% of OneTouch ultra2 values fell within zones C and D, that is with potentially harmful diagnostic and therapeutic decisions.

**Table 3:** Clinical significance of differences in glucose levels between reference and post-intervention values, using the total error allowable.

Glucometer	measurements	n	Mean	SD	% difference	Statistical significance	TEa%	Mean*TEa%	Allowable range		Clinical significance
									min	max	
Accu-check	Reference	91	134.1	84.7	0						
	Cream	91	161.8	98.4	20.6	<0.0001	6.96	9.33	124.8	143.4	Significant
	Reference	91	134.1	84.7	0						
	Cleaning	91	160.8	100.6	19.9	<0.0001	6.96	9.33	124.8	143.4	Significant
	Reference	91	134.1	84.7	0						
	Sanitizer	91	172.1	103.8	28.3	<0.0001	6.96	9.33	124.8	143.4	Significant
	Reference	91	134.1	84.7	0						
	Washing 1	91	150.4	93.8	12.2	<0.0001	6.96	9.33	124.8	143.4	Significant
	Reference	91	134.1	84.7	0						
	Washing 2	91	138.0	86.2	2.9	0.0003	6.96	9.33	124.8	143.4	Non significant
	Reference	91	134.1	84.7	0						
	Washing 3	91	132.4	83.0	-1.3	0.009	6.96	9.33	124.8	143.4	Non signficant
One Touch	Reference	91	138.3	78.8	0						
	Cream	91	179.7	99.7	29.9	<0.0001	6.96	9.63	128.7	147.9	Significant
	Reference	91	138.3	78.8	0						
	Cleaning	91	182.5	102.7	32	<0.0001	6.96	9.63	128.7	147.9	Significant
	Reference	91	138.3	78.8	0						
	Sanitizer	91	202.5	116.7	46.4	<0.0001	6.96	9.63	128.7	147.9	Significant
	Reference	91	138.3	78.8	0						
	Washing 1	91	159.9	97.7	15.6	<0.0001	6.96	9.63	128.7	147.9	Significant
	Reference	91	138.3	78.8	0						
	Washing 2	91	143.0	89.6	3.4	0.057	6.96	9.63	128.7	147.9	Non significant
	Reference	91	138.3	78.8	0						
	Washing 3	91	133.1	78.7	-3.8	<0.0001	6.96	9.63	128.7	147.9	Non significant

SD: Standard deviation, TEa: Total error allowable

### Effects of various interventions in reversing the effect of the cream on capillary glycemia

The mean differences in capillary glucose between values obtained after various interventions and reference values are surmmarised in supplementary Tables 1 and 2. Finger cleaning with wet gauze, application of hand sanitizer and first hand washing did not reverse the effect of hydroquinone on glucose measurements, as the mean differences between these values and the reference were statistically (S1 and S2 Tables) and clinically (Table 3) significant. Instead, application of hand sanitizer caused a further significant increase in capillary glucose. Hand washing led to a gradual attenuation of the effect of hydroquinone on capillary glucose measurements regardless of the glucometer (Fig 1). After two or more hand washings, the mean differences with the reference became clinically non-significant (Table 3), and no value fell within unwanted zones (C, D or E) of the Parke’s consensus grid (figure not shown).

## Discussion

Findings from the current study confirm that hydroquinone-containing creams cause a significant false elevation in capillary glucose levels, and this appears not to be related to the enzyme system of the glucometer used, gender or diabetes status. This false increase is clinically significant and can negatively impact clinical decision, with related potential consequences. Usual interventions such as finger cleaning with wet gauze, or swabbing with sanitizers are ineffective in reversing the effect of hydroquinone-containing cream. Instead, hand sanitizer paradoxically causes a further glycemic increase. Also, washing hands once with soap insufficiently attenuates the effect of the cream. A minimum of two hand washings with soap is required to successfully eliminate the effect of hydroquinone-containing cream on CGM. Altogether, our findings challenge existing practices and suggest novel norms of hand treatment prior to measuring capillary glucose with POC instruments. Also, given the wide over-the-counter use of hydroquinone-containing creams elsewhere, they raise the need for regulatory agencies to require inclusion of all hydroquinone-containing skin formulations as major inteferences in CGM.

The significant increase in capillary glycemia by 28–41 mg/dl after application of the hydroquinone-containing cream in our sample, is in line with the hypothesis that use of creams rich in hydroquinone prior to CGM causes a significant falsely elevated reading. This is true irrespective of glucose measurement principles of the glucometers. Mean differences observed in our study were lower than the 170.3 mg/dl we previously observed in a study performed in 11 healthy volunteers in France [5]. Sobngwi *et al* also reported that hydroquinone-containing creams caused false increase in capillary glycaemia with a mean difference before and after cream application of 213 mg/dl [15]. We attribute the smaller capillary glucose rise observed in our study as compared to previous observations, to the smaller concentration of hydroquinone -thirteen-fold lower (0.155%) than the labelled concentration (2%). Also, though other studies did not calibrate the volume of cream, it may have been higher in their studies. Whether there is a dose-effect relationship between hydroquinone and capillary glycemia is highgly probable, but is still to be investigated.

Finger cleaning, or application of a hand sanitizer did not reverse the effect of the cream. A report on the effects of hand sanitizers on capillary glucose measurement showed that hand sanitizers did not adversely affect test results; however, when exogenous glucose interference was present, the effectiveness of the hand sanitizer on glucose bias depended on the surface area and degree of dilution. The investigators concluded that the use of an instant hand sanitizer may or may not minimize interference from exogenous glucose or interfering substances prior to capillary glucose measurement [16]. Though it was not a major outcome of our study, the paradoxical increase of glycemia after application of hand sanitizer suggest interactions between hydroquinone-containing cream and components of the sanitizer. Careful hand washing following cream application gradually attenuated the effect of hydroquinone-containing creams on capillary glucose levels, with two hand washings appearing as the minimum needed to completely reverse the effects of these creams.

Taken together our findings challenge the effectiveness of common hand treatment practices prior to CGM using glucometers, in removing the effects of exogenous interfering factors on glucose values, particularly in the context of high prevalence of hydroquinone-containing cream use. Typically, those routine practices range from obtaining capillary glucose from fingers without any treatment [1] to variable treatments including finger swabbing with water or sanitizer, or single hand washing [8,9]. But seldom are hands washed repeatedly prior to CGM, as such practice is not currently supported by guidelines [17]. Therefore, in settings where use of hydroquinone-containing creams is frequent, it would be appropriate to enforce the pratice of repeated hand washing prior to measuring capillary glucose with glucometers. This should be complemented by the sensitization of patients who frequently embark on self-monitoring of capillary glucose, on the importance of avoiding or neutralising the effect of extrinsic interfering factors on their capillary glucose values.

The current study focused on the potential effect of one extrinsic interfering factor (hydroquinone), and whether our findings extend to other interfering factors is unknown. However the implications of our findings are rather towards an improvement of current practices, and will in no circumstance lead to further deterioration of blood glucose, should interfering factors other than hydroquinone be involved. We also objectively confirmed the presence and concentration of hydroquinone in the cream used, which was a step forward compared to all previous studies, as these creams, usually from uncontrolled origin, may not contain hydroquinone or may only contain smaller quantities than the indication on the label. The latter was the case in our study and explains the smaller increase in post-cream capillary glucose compared to previous studies. Finally, unlike previous report, we have provided the potential clinical impact of these creams and provided evidence that common practices are ineffective and two hand washings are the likely minimum requirement. Despite the aforementioned strengths, we acknowledge some limitations. Amongst others, calibration of glucometers was done only at the start of the day; a second calibration at the end of the day would have been an absolute confirmation of accuracy of measurements over the day.

## Conclusion

Hydroquinone-containing creams cause a false elevation in capillary glucose level, measured by glucometers that employ either the glucose oxidase or the glucose dehydrogenase enzymatic system. Application of a small volume (1ml) of cream containing very little amount of hydroquinone increases glycemia enough to adversely affect clinical (diagnostic and therapeutic) decisions. Common practices like the use of wet gauze or hand sanitizer do not reverse the effect of the cream, which is only neutralized by a series of at least two hand washings. A systematic practice of washing hands at least twice prior to capillary glucose measurement is likely to translate into more reliable test results.

## Supporting information

S1 Dataset Hyroquinone and capillary glycemia data. (XLSX)

S1 Table Glucose measurements after various intervention using the Accu-Check active glucometer. (DOCX)

S2 Table Glucose measurements after various interventions using the one touch ultra glucometer. (DOCX)

S1 Fig Distribution of interventions and capillary glucose measurements between fingers. G0: reference capillary glucose, G1: capillary glucose after cream application; G2A: capillary glucose after finger swabbing with soaked gauze; G2B: capillary glucose after application of hand sanitizer; G3A, 3B, 3C: capillary glucose after first, second and third hand washings, respectively.
(DOCX)

## References

[1] Rosindale S, Bower L, Farleigh E, Francis M, Drew C, Cooke P. A community study of accuracy of blood glucose meter results. J Diabetes Nurs. 2004;8(4):272–6.

[2] Rajaratnam H, Pathmanathan S. How reliable are capillary blood glucose measurements? Sri Lanka J Diabetes Endocrinol Metab. 2012 3 25;1(1):22.

[3] Ginsberg BH. Factors Affecting Blood Glucose Monitoring: Sources of Errors in Measurement. J Diabetes Sci Technol Online. 2009 7;3(4):903–13. 10.1177/193229680900300438 PMC2769960 20144340

[4] Olumide YM, Akinkugbe AO, Altraide D, Mohammed T, Ahamefule N, Ayanlowo S, et al Complications of chronic use of skin lightening cosmetics. Int J Dermatol. 2008 4;47(4):344–53. 10.1111/j.1365-4632.2008.02719.x 18377596

[5] Bouché CH, Garnier J-P, Choukem S-P, Gautier J-F. Falsely elevated capillary glucose and ketone levels and use of skin lightening creams. The BMJ. 2015 7 29;351:h3879 10.1136/bmj.h3879 26223684

[6] Bihan H, Fysekidis M, Harbuz V, Reach G, Cohen R. False-Positive Blood Glucose and Ketone Values With Lightening Cream. Ann Intern Med. 2011 11 1;155(9):649. 10.7326/0003-4819-155-9-201111010-00023 22041962

[7] Sobngwi E, Omengue A, Bissek A-C, Ama V, Mbanya J-C, Gautier J-F. O7 Effets des laits corporels sur la mesure de la glycémie capillaire. Diabetes Metab. 2013 3 29;39, Supplement 1:A2.

[8] Hortensius J, van der Bijl JJ, Kleefstra N, Houweling ST, Bilo HJG. Self-Monitoring of Blood Glucose: Professional Advice and Daily Practice of Patients With Diabetes. Diabetes Educ. 2012 1 1;38(1):101–7. 10.1177/0145721711427787 22146788

[9] Hortensius J, Slingerland RJ, Kleefstra N, Logtenberg SJJ, Groenier KH, Houweling ST, et al Self-Monitoring of Blood Glucose: The Use of the First or the Second Drop of Blood. Diabetes Care. 2011 2 2;34(3):556–60. 10.2337/dc10-1694 21289231 PMC3041180

[10] ISO Standards for Blood Glucose Meters and Test Strips [Internet]. [cited 2018 Mar 14]. https://www.diabetes.co.uk/blood-glucose-meters/iso-accuracy-standards.html

[11] García PL, Santoro MIRM, Singh AK, Kedor-Hackmann ERM. Determination of optimum wavelength and derivative order in spectrophotometry for quantitation of hydroquinone in creams. Rev Bras Ciênc Farm. 2007 9;43(3):397–404.

[12] Parkes JL, Slatin SL, Pardo S, Ginsberg BH. A new consensus error grid to evaluate the clinical significance of inaccuracies in the measurement of blood glucose. Diabetes Care. 2000 8 1;23(8):1143–8. 10937512 10.2337/diacare.23.8.1143

[13] Harr KE, Flatland B, Nabity M, Freeman KP, ASVCP. ASVCP guidelines: allowable total error guidelines for biochemistry. Vet Clin Pathol Am Soc Vet Clin Pathol. 2013 12;42(4):424–36. 10.1111/vcp.12101 24320779

[14] Desirable Biological Variation Database specifications—Westgard [Internet]. [cited 2018 May 28]. https://www.westgard.com/biodatabase1.htm

[15] Sobngwi E, Omengue A, Bissek A-C, Ama V, Mbanya J-C, Gautier J-F. O7 Effets des laits corporels sur la mesure de la glycémie capillaire. Diabetes Metab. 2013 3;39:A2.

[16] Mahoney JJ, Ellison JM, Glaeser D, Price D. The effect of an instant hand sanitizer on blood glucose monitoring results. J Diabetes Sci Technol. 2011 11;5(6):1444–8. 10.1177/193229681100500616 22226262 PMC3262711

[17] Resources and tools [Internet]. [cited 2018 May 28]. https://www.idf.org/our-activities/advocacy-awareness/resources-and-tools/85:self-monitoring-of-blood-glucose-in-non-insulin-treated-type-2-diabetes.html

