# Supplementary material for: Effects of hydroquinone-containing creams on capillary glycemia before and after serial hand washings in Africans
Source: PLoS One. 2018 Aug 28;13(8):e0202271. doi: 10.1371/journal.pone.0202271 (PMC6112636; doi:10.1371/journal.pone.0202271)
Supplement: S1 Table — (DOCX) [file pone.0202271.s002.docx]

**Supplementary table 1: Glucose measurements after various intervention using the Accu-Check active glucometer**

| **Variables** | **Total** | **Men** | **Women** | **p-value** |  | **No diabetes** | **Diabetes** | **p-value** |
| --- | --- | --- | --- | --- | --- | --- | --- | --- |
| **After hand wash (reference)** |  |  |  |  |  |  |  |  |
| Mean (SD) | 134 (85) | 131 (74) | 137 (94) | 0.733 |  | 85 (13) | 205 (95) | <0.0001 |
| Median (min-max) | 95 (50-452) | 94 (50-302) | 99 (80-135) | 0.774 |  | 84 (50-113) | 226 (88-452) | <0.0001 |
| Shapiro p | <0.0001 | <0.0001 | <0.0001 |  |  | 0.206 | 0.003 |  |
| Skewness (p Agostino test) | 1.750 (<0.0001) | 1.074 (0.004) | 1.984 (<0.0001) |  |  | -0.209 (0.492) | 0.692 (0.064) |  |
| Kurtosis (p Anscombe-Glynn test) | 5.707 (0.001) | 2.553 (0.692) | 6.304 (0.002) |  |  | 3.255 (0.420) | 3.328 (0.425) |  |
| Coefficient of variation (%) | 63.1 | 56.8 | 68.6 |  |  | 15.3 | 46.1 |  |
| **Cream application** |  |  |  |  |  |  |  |  |
| Mean (SD) | 162 (98) | 160 (78) | 163 (115) | 0.906 |  | 116 (44) | 229 (116) | <0.0001 |
| Median (min-max) | 119 (79-565) | 120 (80-315) | 119 (79-565) | 0.691 |  | 97 (79-315) | 246 (82-565) | <0.0001 |
| Shapiro p | <0.0001 | <0.0001 | <0.0001 |  |  | <0.0001 | 0.0005 |  |
| Skewness (p Agostino test) | 1.887 (<0.0001) | 0.583 (0.090) | 2.143 (<0.0001) |  |  | 2.260 (<0.0001) | 1.091 (0.006) |  |
| Kurtosis (p Anscombe-Glynn test) | 7.273 (<0.0001) | 1.738 (0.0004) | 7.304 (0.0007) |  |  | 9.028 (<0.0001) | 4.507 (0.049) |  |
| Coefficient of variation (%) | 60.8 | 48.8 | 70.5 |  |  | 38.3 | 50.9 |  |
| **Reference vs cream** |  |  |  |  |  |  |  |  |
| Mean (95%CI), Ref-Cream | -28 [-37 to -18] | -30 [-45 to -14] | -26 [-38 to -14] | 0.701 |  | -30 [-43 to -18] | -23 [-38 to -9] | 0.566 |
| Paired t-test | <0.0001 | <0.0001 | <0.0001 |  |  | <0.0001 | 0.002 |  |
| Pearson correlation (95% CI) | 0.891 (0.840-0.927) | 0.789 (0.642-0.880) | 0.947 (0.906-0.970) |  |  | 0.054 (-0.217 to 0.317) | 0.936 (0.877-0.967) |  |
| Spearman correlation | 0.747 | 0.663 | 0.820 |  |  | 0.375 | 0.886 |  |
| **Cleaning** |  |  |  |  |  |  |  |  |
| Mean (SD) | 161 (101) | 160 (81) | 161 (117) | 0.940 |  | 114 (44) | 229 (119) | <0.0001 |
| Median (min-max) | 110 (74-590) | 114 (74-306) | 110 (76-590) | 0.933 |  | 98 (74-300) | 230 (89-590) | <0.0001 |
| Shapiro p | <0.0001 | <0.0001 | <0.0001 |  |  | <0.0001 | 0.0003 |  |
| Skewness (p Agostino test) | 2.035 (<0.0001) | 0.601 (0.081) | 2.354 (<0.0001) |  |  | 2.103 (<0.0001) | 1.297 (0.003) |  |
| Kurtosis (p Anscombe-Glynn test) | 8.204 (<0.0001 | 1.827 (0.003) | 8.446 (0.0002) |  |  | 7.708 (0.0003 | 5.125 (0.019) |  |
| Coefficient of variation (%) | 62.6 | 50.6 | 72.4 |  |  | 39.0 | 52.1 |  |
| **Reference vs. cleaning** |  |  |  |  |  |  |  |  |
| Mean (95%CI), Ref-cleaning | -27 [-36 to -17] | -29 [-44 to -14] | -24 [-37 to -12] | 0.641 |  | -28 [-41 to -16] | -24 [-39 to -9] | 0.674 |
| Paired t-test | <0.0001 | 0.0003 | 0.0003 |  |  | <0.0001 | 0.002 |  |
| Pearson correlation (95% CI) | 0.893 (0.842-0.928) | 0.804 (0.667-0.889) | 0.942 (0.897-0.967) |  |  | -0.043 (-0.307 to 0.228) | 0.940 (0.887-0.969) |  |
| Spearman correlation | 0.720 | 0.668 | 0.796 |  |  | 0.248 | 0.912 |  |
| **Sanitizer** |  |  |  |  |  |  |  |  |
| Mean (SD) | 172 (104) | 171 (78) | 173 (124) | 0.901 |  | 125 (46) | 241 (124) | <0.0001 |
| Median (min-max) | 132 [79-595] | 141 (80-315) | 115 (79-595) | 0.475 |  | 105 (79-234) | 240 (94-595) | <0.0001 |
| Shapiro p | <0.0001 | 0.0002 | <0.0001 |  |  | <0.0001 | 0.0003 |  |
| Skewness (p Agostino test) | 1.966 (<0.0001) | 0.463 (0.170) | 2.140 (<0.0001) |  |  | 1.145 (0.001) | 1.228 (0.003) |  |
| Kurtosis (p Anscombe-Glynn test) | 7.800 (<0.001 | 1.736 (0.0004) | 7.230 (0.0008) |  |  | 2.856 (0.880) | 4.619 (0.041) |  |
| Coefficient of variation (%) | 60.3 | 45.8 | 71.5 |  |  | 36.9 | 51.7 |  |
| **Reference vs sanitizer** |  |  |  |  |  |  |  |  |
| Mean (95%CI), Ref-sanitizer | -38 [-49 to -27] | -40 [-54 to -25] | -36 [-0.53 to -20] | 0.764 |  | -39 [-52 to -26] | -36 [-56 to -16] | 0.789 |
| Paired t-test | <0.0001 | <0.0001 | <0.0001 |  |  | <0.0001 | 0.0007 |  |
| Pearson correlation (95% CI) | 0.862 (0.797-0.907) | 0.800 (0.656-0.885) | 0.899 (0.824-0.943) |  |  | -0.041 (-0.305 to 0.229) | 0.888 (0.791-0.941) |  |
| Spearman correlation | 0.689 | 0.654 | 0.732 |  |  | 0.181 | 0.862 |  |
| **One washing** |  |  |  |  |  |  |  |  |
| Mean (SD) | 150 (94) | 147 (72) | 153 (111) | 0.755 |  | 103 (27) | 219 (113) | <0.0001 |
| Median (min-max) | 105 [75-565] | 107 (80-324) | 103 (75-565) | 0.556 |  | 96 (75-212) | 221 (87-565) | <0.0001 |
| Shapiro p | <0.0001 | <0.0001 | <0.0001 |  |  | <0.0001 | 0.0002 |  |
| Skewness (p Agostino test) | 2.260 (<0.0001) | 1.009 (0.006) | 2.374 (<0.0001) |  |  | 2.365 (<0.0001) | 1.352 (0.001) |  |
| Kurtosis (p Anscombe-Glynn test) | 9.224 (<0.0001) | 2.658 (0.866) | 8.610 (0.0002) |  |  | 8.626 (0.0001) | 5.325 (0.014) |  |
| Coefficient of variation (%) | 62.4 | 49.0 | 72.4 |  |  | 25.8 | 51.4 |  |
| **Reference vs washing 1** |  |  |  |  |  |  |  |  |
| Mean (95%CI), Ref-washing 1 | -16 [-22 to -10] | -16 [-25 to -7] | -16 [-25 to -7] | 0.994 |  | -18 [-25 to -10] | -14 [-25 to -4] | 0.613 |
| Paired t-test | <0.0001 | 0.0007 | 0.0005 |  |  | <0.0001 | 0.008 |  |
| Pearson correlation (95% CI) | 0.950 (0.925-0.967) | 0.919 (0.855-0.955) | 0.971 (0.948-0.984) |  |  | 0.071 (-0.201 to 0.332) | 0.970 (0.941-0.984) |  |
| Spearman correlation | 0.795 | 0.734 | 0.852 |  |  | 0.388 | 0.945 |  |
| **Two washings** |  |  |  |  |  |  |  |  |
| Mean (SD) | 138 (86) | 135 (70) | 141 (99) | 0.754 |  | 90 (12) | 208 (99) | <0.0001 |
| Median (min-max) | 100 [62-495] | 101 (62-305) | 99 (68-495) | 0.685 |  | 89 (62-119) | 218 (87-495) | <0.0001 |
| Shapiro p | <0.0001 | <0.0001 | <0.0001 |  |  | 0.879 | 0.001 |  |
| Skewness (p Agostino test) | 2.053 (<0.0001) | 1.143 (0.003) | 2.205 (<0.0001) |  |  | 0.125 (0.679) | 1.064 (0.008) |  |
| Kurtosis (p Anscombe-Glynn test) | 7.552 (<0.0001) | 2.765 (0.968) | 7.551 (0.0005) |  |  | 2.744 (0.942) | 4.362 (0.062) |  |
| Coefficient of variation (%) | 62.4 | 52.0 | 70.7 |  |  | 13.0 | 47.8 |  |
| **Reference vs washing 2** |  |  |  |  |  |  |  |  |
| Mean (95%CI), Ref-washing 2 | -4 [-6 to -2] | -4 [-7 to -1] | -4 [-7 to -1] | 0.846 |  | -5 [-7 to -2] | -3 [-7 to 1] | 0.434 |
| Paired t-test | 0.0003 | 0.009 | 0.016 |  |  | <0.0001 | 0.167 |  |
| Pearson correlation (95% CI) | 0.993 (0.990-0.995) | 0.992 (0.986-0.996) | 0.996 (0.993-0.998) |  |  | 0.800 (0.677-0.879) | 0.993 (0.986-0.996) |  |
| Spearman correlation | 0.948 | 0.910 | 0.976 |  |  | 0.824 | 0.981 |  |
| **Three washings** |  |  |  |  |  |  |  |  |
| Mean (SD) | 132 (83) | 129 (70) | 136 (94) | 0.700 |  | 85 (11) | 201 (94) | <0.0001 |
| Median (min-max) | 95 [53-455] | 94 (53-300) | 95 (66-455) | 0.994 |  | 86 (53-107) | 216 (85-455) | <0.0001 |
| Shapiro p | <0.0001 | <0.0001 | <0.0001 |  |  | 0.553 | 0.003 |  |
| Skewness (p Agostino test) | 1.875 (<0.0001) | 1.144 (0.003) | 2.048 (<0.0001) |  |  | -0.412 (0.182) | 0.828 (0.030) |  |
| Kurtosis (p Anscombe-Glynn test) | 6.361 (0.0004) | 2.797 (0.921) | 6.613 (0.002) |  |  | 3.046 (0.626) | 3.553 (0.246) |  |
| Coefficient of variation (%) | 62.7 | 54.6 | 69.2 |  |  | 13.4 | 47.0 |  |
| **Reference vs washing 3** |  |  |  |  |  |  |  |  |
| Mean (95%CI), Ref-washing 3 | 2 [0.4 to 3] | 2 [-0.2 to 4] | 1 [0 to 3] | 0.638 |  | 0 [-1 to 1] | 4 [2 to 6] | 0.0009 |
| Paired t-test | 0.009 | 0.069 | 0.057 |  |  | 0.981 | 0.0002 |  |
| Pearson correlation (95% CI) | 0.997 (0.996-0.998) | 0.996 (0.993-0.998) | 0.998 (0.997-0.999) |  |  | 0.904 (0.839-0.943) | 0.998 (0.996-0.999) |  |
| Spearman correlation | 0.967 | 0.955 | 0.975 |  |  | 0.880 | 0.994 |  |
