# Supplementary material for: Effects of hydroquinone-containing creams on capillary glycemia before and after serial hand washings in Africans
Source: PLoS One. 2018 Aug 28;13(8):e0202271. doi: 10.1371/journal.pone.0202271 (PMC6112636; doi:10.1371/journal.pone.0202271)
Supplement: S2 Table — (DOCX) [file pone.0202271.s003.docx]

**Supplementary table 2: Glucose measurements after various interventions using the One Touch Ultra® glucometer**

| **Variables** | **Total** | **Men** | **Women** | **p-value** |  | **No diabetes** | **Diabetes** | **p-value** |
| --- | --- | --- | --- | --- | --- | --- | --- | --- |
| **After hand wash (reference)** |  |  |  |  |  |  |  |  |
| Mean (SD) | 138 (79) | 136 (72) | 141 (85) | 0.757 |  | 90 (13) | 209 (81) | <0.0001 |
| Median (min-max) | 100 [51-409] | 99 (51-297) | 102 (69-409) | 0.608 |  | 91 (51-115) | 216 (96-409) | <0.0001 |
| Shapiro p | <0.0001 | <0.0001 | <0.0001 |  |  | 0.281 | 0.034 |  |
| Skewness (p Agostino test) | 1.467 (<0.0001) | 0.976 (0.008) | 1.706 (<0.0001) |  |  | -0.592 (0.062) | 0.417 (0.247) |  |
| Kurtosis (p Anscombe-Glynn test) | 4.427 (<0.0001) | 2.364 (0.388) | 5.144 (0.0134) |  |  | 3.410 (0.309) | 2.857 (0.800) |  |
| Coefficient of variation (%) | 57.0 | 53.4 | 60.5 |  |  | 14.4 | 39.0 |  |
| **Cream application** |  |  |  |  |  |  |  |  |
| Mean (SD) | 180 (100) | 166 (76) | 193 (117) | 0.196 |  | 139 (56) | 239 (118) | <0.0001 |
| Median (min-max) | 157 (73-585) | 142 (73-358) | 157 (77-585) | 0.317 |  | 113 (73-269) | 232 (78-585) | <0.0001 |
| Shapiro p | <0.0001 | 0.001 | <0.0001 |  |  | <0.0001 | 0.002 |  |
| Skewness (p Agostino test) | 1.696 (<0.0001) | 0.633 (0.068) | 1.710 (<0.0001) |  |  | 0.860 (0.010) | 1.165 (0.004) |  |
| Kurtosis (p Anscombe-Glynn test) | 6.925 (0.0001) | 2.358 (0.380) | 6.035 (0.004) |  |  | 2.338 (0.269) | 4.587 (0.043) |  |
| Coefficient of variation (%) | 55.5 | 45.9 | 60.7 |  |  | 40.6 | 49.5 |  |
| **Reference vs cream** |  |  |  |  |  |  |  |  |
| Mean (95%CI), Ref-Cream | -41 [-53 to -30] | -30 [-45 to -16] | -51 [-70 to -34] | 0.062 |  | -49 [-64 to -34] | -30 [-0.48 to -12] | 0.114 |
| Paired t-test | <0.0001 | 0.0001 | <0.0001 |  |  | <0.0001 | 0.002 |  |
| Pearson correlation (95% CI) | 0.832 (0.755-0.886) | 0.798 (0.657-0.885) | 0.865 (0.769-0.923) |  |  | 0.181 (-0.091 to 0.428) | 0.919 (0.847-0.958) |  |
| Spearman correlation | 0.677 | 0.679 | 0.649 |  |  | 0.328 | 0.896 |  |
| **Cleaning** |  |  |  |  |  |  |  |  |
| Mean (SD) | 182 (103) | 177 (84) | 188 (118) | 0.617 |  | 139 (56) | 239 (118) | <0.0001 |
| Median (min-max) | 140 [77-592] | 159 (77-335) | 139 (85-592) | 0.760 |  | 119 (77-243) | 279 (90-592) | <0.0001 |
| Shapiro p | <0.0001 | 0.0004 | <0.0001 |  |  | <0.0001 | 0.001 |  |
| Skewness (p Agostino test) | 1.647 (<0.0001) | 0.446 (0.186) | 1.907 (<0.0001) |  |  | 0.836 (0.012) | 0.897 (0.020) |  |
| Kurtosis (p Anscombe-Glynn test) | 6.453 (0.0003) | 1.767 (0.0009) | 6.509 (0.002) |  |  | 2.426 (0.398) | 4.124 (0.092) |  |
| Coefficient of variation (%) | 56.3 | 47.5 | 63.0 |  |  | 35.3 | 47.5 |  |
| **Reference vs cleaning** |  |  |  |  |  |  |  |  |
| Mean (95%CI), Ref-cleaning | -44 [-55 to -34] | -41 [-56 to -26] | -47 [-62 to -31] | 0.603 |  | -44 [-57 to -31] | -44 [-63 to -26] | 0.996 |
| Paired t-test | <0.0001 | <0.0001 | <0.0001 |  |  | <0.0001 | <0.0001 |  |
| Pearson correlation (95% CI) | 0.874 (0.815-0.915) | 0.811 (0.677-0.893) | 0.915 (0.852-0.952) |  |  | 0.047 (-0.224 to 0.310) | 0.920 (0.849-0.958) |  |
| Spearman correlation | 0.662 | 0.633 | 0.690 |  |  | 0.203 | 0.833 |  |
| **Sanitizer** |  |  |  |  |  |  |  |  |
| Mean (SD) | 202 (117) | 214 (112) | 192 (121) | 0.366 |  | 159 (98) | 266 (114) | <0.0001 |
| Median (min-max) | 178 (83-605) | 196 (90-593) | 150 (83-605) | 0.094 |  | 121 (83-593) | 275 (110-605) | <0.0001 |
| Shapiro p | <0.0001 | <0.0001 | <0.0001 |  |  | <0.0001 | 0.0002 |  |
| Skewness (p Agostino test) | 1.812 (<0.0001) | 1.664 (<0.0001) | 1.995 (<0.0001) |  |  | 3.259 (<0.0001) | 1.324 (0.002) |  |
| Kurtosis (p Anscombe-Glynn test) | 6.609 (0.0002) | 6.515 (0.002) | 6.957 (0.001) |  |  | 14.825 (<0.0001) | 5.227 (0.016) |  |
| Coefficient of variation (%) | 57.6 | 52.2 | 63.3 |  |  | 61.3 | 43.0 |  |
| **Reference vs sanitizer** |  |  |  |  |  |  |  |  |
| Mean (95%CI), Ref-sanitizer | -64 [-82 to -46] | -78 [-110 to -47] | -51 [-71 to -31] | 0.137 |  | -69 [-95 to -43] | -57 [-81 to -33] | 0.521 |
| Paired t-test | <0.0001 | <0.0001 | <0.0001 |  |  | <0.0001 | <0.0001 |  |
| Pearson correlation (95% CI) | 0.664 (0.530-0.765) | 0.429 (0.152-0.644) | 0.847 (0.740-0.912) |  |  | 0.144 (-0.129 to 0.396) | 0.776 (0.604-0.879) |  |
| Spearman correlation | 0.628 | 0.664 | 0.628 |  |  | 0.159 | 0.691 |  |
| **One washing** |  |  |  |  |  |  |  |  |
| Mean (SD) | 160 (98) | 155 (76) | 164 (115) | 0.640 |  | 108 (24) | 236 (114) | <0.0001 |
| Median (min-max) | 119 [66-590] | 117 (77-297) | 121 (66-590) | 0.760 |  | 104 (66-180) | 238 (94-590) | <0.0001 |
| Shapiro p | <0.0001 | <0.0001 | <0.0001 |  |  | 0.0009 | 0.0002 |  |
| Skewness (p Agostino test) | 2.233 (<0.0001) | 0.822 (0.022) | 2.410 (<0.0001) |  |  | 1.152 (0.001) | 1.420 (0.009) |  |
| Kurtosis (p Anscombe-Glynn test) | 9.215 (<0.0001) | 2.094 (0.086) | 8.793 (0.0001) |  |  | 4.693 (0.025) | 5.694 (0.008) |  |
| Coefficient of variation (%) | 61.1 | 49.2 | 69.8 |  |  | 22.0 | 48.3 |  |
| **Reference vs washing 1** |  |  |  |  |  |  |  |  |
| Mean (95%CI), Ref-washing 1 | -22 [-30 to -13] | -19 [-28 to -11] | -24 [-38 to -9] | 0.603 |  | -18 [-24 to -11] | -27 [-46 to -8] | 0.280 |
| Paired t-test | <0.0001 | <0.0001 | 0.002 |  |  | <0.0001 | 0.006 |  |
| Pearson correlation (95% CI) | 0.918 (0.879-0.946) | 0.931 (0.877-0.962) | 0.922 (0.864-0.956) |  |  | 0.311 (0.048-0.535) | 0.886 (0.789-0.940) |  |
| Spearman correlation | 0.788 | 0.826 | 0.739 |  |  | 0.437 | 0.843 |  |
| **Two washings** |  |  |  |  |  |  |  |  |
| Mean (SD) | 143 (90) | 140 (76) | 145 (102) | 0.801 |  | 91 (15) | 219 (98) | <0.0001 |
| Median (min-max) | 103 [54-525] | 107 (54-285) | 103 (65-525) | 0.911 |  | 90 (54-115) | 220 (94-525) | <0.0001 |
| Shapiro p | <0.0001 | <0.0001 | <0.0001 |  |  | 0.111 | 0.0002 |  |
| Skewness (p Agostino test) | 2.043 (<0.0001) | 0.884 (0.015) | 2.377 (<0.0001) |  |  | -0.433 (0.163) | 1.352 (0.001) |  |
| Kurtosis (p Anscombe-Glynn test) | 8.063 (<0.0001) | 2.233 (0.213) | 8.774 (0.0001) |  |  | 2.716 (0.896) | 5.671 (0.008) |  |
| Coefficient of variation (%) | 62.7 | 53.8 | 70.0 |  |  | 16.4 | 44.7 |  |
| **Reference vs washing 2** |  |  |  |  |  |  |  |  |
| Mean (95%CI), Ref-washing 2 | -5 [-9 to 0.1] | -5 [-11 to 1] | -4 [-12 to 3] | 0.935 |  | -1 [-3 to 2] | -10 [-22 to 1] | 0.046 |
| Paired t-test | 0.057 | 0.108 | 0.245 |  |  | 0.628 | 0.064 |  |
| Pearson correlation (95% CI) | 0.970 (0.955-0.980) | 0.965 (0.937-0.981) | 0.976 (0.958-0.987) |  |  | 0.731 (0.576-0.835) | 0.947 (0.898-0.972) |  |
| Spearman correlation | 0.900 | 0.909 | 0.897 |  |  | 0.696 | 0.922 |  |
| **Three washings** |  |  |  |  |  |  |  |  |
| Mean (SD) | 133 (79) | 131 (73) | 135 (84) | 0.808 |  | 85 (12) | 204 (82) | <0.0001 |
| Median (min-max) | 98 [54-405] | 96 (54-284) | 98 (65-405) | 0.796 |  | 84 (54-106) | 216 (92-405) | <0.0001 |
| Shapiro p | <0.0001 | <0.0001 | <0.0001 |  |  | 0.206 | 0.012 |  |
| Skewness (p Agostino test) | 1.475 (<0.0001) | 0.994 (0.007) | 1.736 (<0.0001) |  |  | -0.245 (0.420) | 0.418 (0.246) |  |
| Kurtosis (p Anscombe-Glynn test) | 4.442 (0.021) | 2.355 (0.376) | 5.311 (0.011) |  |  | 2.794 (0.976) | 2.858 (0.800) |  |
| Coefficient of variation (%) | 59.1 | 55.8 | 62.4 |  |  | 14.6 | 40.0 |  |
| **Reference vs washing 3** |  |  |  |  |  |  |  |  |
| Mean (95%CI), Ref-washing 3 | 5 [3-8] | 5 [0.4 to 9] | 6 [3 to 8] | 0.648 |  | 5 [3 to 7] | 5 [0.2 to 10] | 0.925 |
| Paired t-test | <0.0001 | 0.034 | <0.0001 |  |  | <0.0001 | 0.043 |  |
| Pearson correlation (95% CI) | 0.989 (0.984-0.993) | 0.982 (0.966-0.990) | 0.995 (0.990-0.997) |  |  | 0.801 (0.678-0.880) | 0.982 (0.965-0.991) |  |
| Spearman correlation | 0.925 | 0.927 | 0.922 |  |  | 0.743 | 0.954 |  |
