# Supplementary material for: Effects of hydroquinone-containing creams on capillary glycemia before and after serial hand washings in Africans
Source: PLoS One. 2018 Aug 28;13(8):e0202271. doi: 10.1371/journal.pone.0202271 (PMC6112636; doi:10.1371/journal.pone.0202271)
Supplement: S1 Fig — G0: reference capillary glucose, G1: capillary glucose after cream application; G2A: capillary glucose after finger swabbing with soaked gauze; G2B: capillary glucose after application of hand sanitizer; G3A, 3B, 3C: capillary glucose after first, second and third hand washings, respectively. (DOCX) [file pone.0202271.s004.docx]

**Supplementary figure**: Distribution of interventions and capillary glucose measurements between fingers. G0: reference capillary glucose, G1: capillary glucose after cream application; G2A: capillary glucose after finger swabbing with soaked gauze; G2B: capillary glucose after application of hand sanitizer; G3A, 3B, 3C: capillary glucose after first, second and third hand washings, respectively.
